# Mesenteric neurofibromatosis complicated by acute appendicitis: case report and review of the literature

**DOI:** 10.3389/fonc.2026.1696267

**Published:** 2026-05-20

**Authors:** Jixin Fu, Junguang Liu, Xin Sui

**Affiliations:** 1 Department of Gastrointestinal Surgery, Weihai Central Hospital Affiliated to Qingdao University, Weihai Central Hospital, Weihai, Shandong, China; 2 Pediatric Surgery, Weihai Central Hospital Affiliated to Qingdao University, Weihai Central Hospital, Weihai, Shandong, China

**Keywords:** acute appendicitis, case report, laparoscopic surgery, mesenteric neurofibromatosis, neurofibromatosis type 1

## Abstract

**Background:**

Mesenteric neurofibromatosis, a rare complication of neurofibromatosis type 1 (NF-1), comprises benign plexiform neurofibromas with recognized potential for malignant transformation. These lesions are frequently asymptomatic and diagnostically elusive, mimicking more common gastrointestinal pathologies. We report the incidental intraoperative discovery of mesenteric neurofibromatosis during surgery for acute appendicitis in an NF-1 patient, a presentation scarcely documented previously. This case highlights the occult nature of gastrointestinal involvement in NF-1 and underscores the critical need for systematic imaging surveillance to enable early detection and intervention.

**Case presentation:**

A 17-year-old female presented with a one-week history of right lower quadrant pain. An abdominal CT scan was consistent with acute appendicitis. Physical examination revealed numerous café-au-lait macules, and a significant family history confirmed a concurrent diagnosis of neurofibromatosis type 1 (NF-1). During a scheduled laparoscopic appendectomy, diffuse nodular lesions were incidentally discovered throughout the small bowel mesentery. Histopathological analysis of a biopsied lesion confirmed mesenteric neurofibromatosis. The patient had an uncomplicated postoperative recovery and remained asymptomatic at three-month follow-up.

**Conclusion:**

These findings underscore the necessity for imaging-based screening to detect gastrointestinal lesions early in patients with neurofibromatosis type 1 (NF-1). Heightened awareness of the potential presence of mesenteric neurofibromatosis, a rare manifestation of NF-1, is particularly warranted in the surgical setting when abdominal procedures are indicated for these patients.

## 1 Introduction

Neurofibromatosis type 1 (NF-1) is the most common multi-organ genetic disorder, characterized by the development of tumors related to the nervous system. This disease is caused by mutations in the gene encoding neurofibromin, an important protein involved in cellular signaling and growth regulation, located on the long arm of chromosome 17 (1–4). Mutations in the NF1 gene result in abnormal signaling within the Ras oncogene pathway, and the resulting reduction in negative regulatory function leads to increased cell proliferation (5). The incidence rate is approximately 0.033%, and the prevalence is similar across all races and genders. Neurological, skeletal, and skin abnormalities are among the typical symptoms of this disease (1–3, 6, 7). Gastrointestinal involvement in NF-1 most commonly manifests as plexiform neurofibromas (PNs), which are benign peripheral nerve sheath tumors arising from multiple nerve fascicles (8). When these lesions diffusely involve the mesentery, the condition is termed mesenteric neurofibromatosis.

Classic clinical manifestations of NF1 include benign soft tissue tumors of the skin (neurofibromas) and various neurocutaneous features, such as café-au-lait spots and Lisch nodules. However, the disease has a multisystemic presentation, with gastrointestinal (GI) involvement occurring in 10-25% of cases, but GI manifestations of NF-1 are often nonspecific, leading to frequent misdiagnosis in clinical practice (9). Mesenteric involvement in neurofibromatosis is rare in both adults and children, with only case reports describing such presentations (6). The clinical presentation of mesenteric neurofibromatosis may be asymptomatic or accompanied by various gastrointestinal symptoms, including abdominal pain, weight loss, diarrhea, ulcers, intestinal obstruction, intussusception, or intestinal torsion, which are nonspecific and easily confused with other digestive system diseases (10). The disease is typically benign, with only a small number of cases progressing to malignancy. Therefore, early diagnosis is crucial for improving treatment outcomes.

This paper reports a case of a 15-year-old female patient who was incidentally found to have diffuse mesenteric nodules during laparoscopic appendectomy. Pathological examination of mesenteric biopsy confirmed the diagnosis of neurofibromatosis. This case adds a new example to the literature on this rare disease.

## 2 Case presentation

A 15-year-old female presented to the emergency department in May 2025 with a one-week history of recurrent, dull right lower abdominal pain, accompanied by intermittent exacerbations. She denied nausea, vomiting, diarrhea, abdominal distension, or fever. An initial ultrasound performed at an outside facility was suggestive of acute appendicitis. The patient experienced temporary symptomatic improvement following a course of ceftriaxone, but her symptoms recurred promptly upon discontinuation of antibiotics.

**Figure 1:**
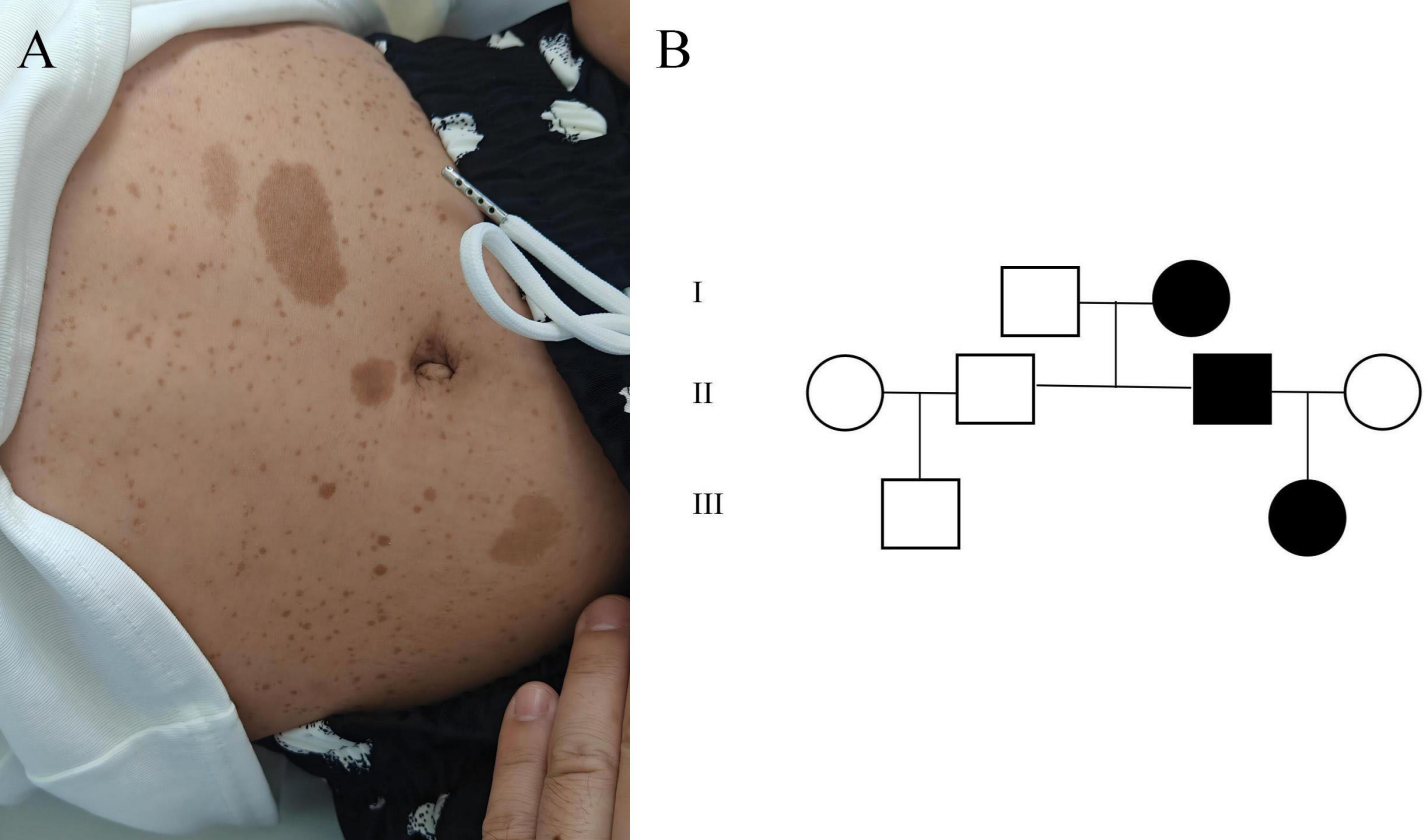
**(A)** revealed multiple café-au-lait macules of varying sizes distributed across the patient’s trunk and extremities; **(B)** shows the genetic pedigree of the patient’s family.

Physical examination revealed multiple café-au-lait macules of varying sizes distributed across the patient’s trunk and extremities (**Figure 1A**). Similar cutaneous findings were observed in the patient’s father. Further family history investigation confirmed an autosomal dominant pattern consistent with neurofibromatosis type 1, as detailed in the genetic pedigree (**Figure 1B**).

On physical examination, the abdomen was soft and non-distended, with extensive café-au-lait macules evident across the surface. There was no visible peristalsis, distension, or signs of obstruction. Palpation revealed tenderness localized to the right lower quadrant, with mild rebound tenderness. No palpable masses, organomegaly, or hernias were detected. Bowel sounds were normoactive.

**Figure 2:**
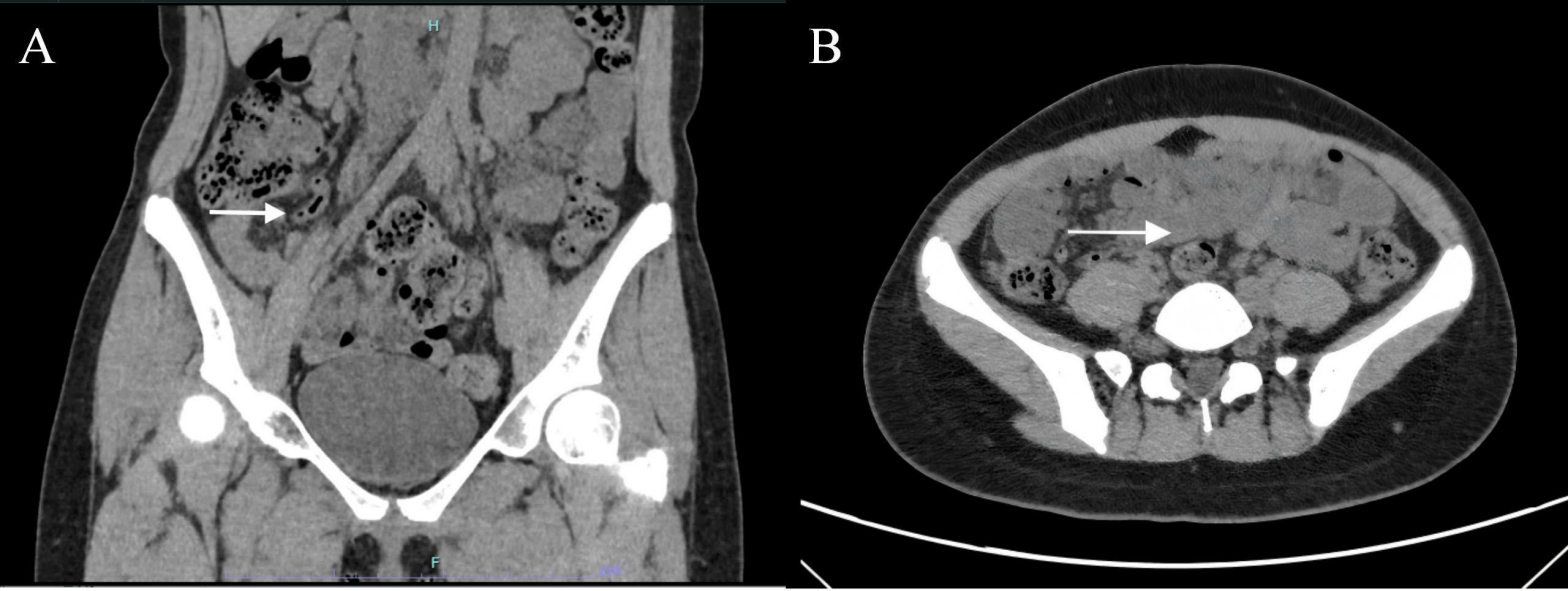
**(A)** Abdominal CT imaging demonstrated appendiceal wall thickening, luminal gas, and peri-appendiceal fat stranding, accompanied by multiple enlarged lymph nodes in the ileocecal region (white arrow); **(B)** Abdominal CT revealed nodular mesenteric masses (white arrow).

Laboratory studies revealed leukocytosis (11.6×10^9^/L) with neutrophilia (82.6%), consistent with an acute inflammatory process. Hemoglobin and platelet counts were within normal limits. Abdominal CT imaging demonstrated appendiceal wall thickening, luminal gas, and peri-appendiceal fat stranding, accompanied by multiple enlarged lymph nodes in the ileocecal region (**Figure 2A**). Subsequent gastroscopy findings were consistent with chronic superficial gastritis, while colonoscopy was unremarkable.

**Figure 3:**
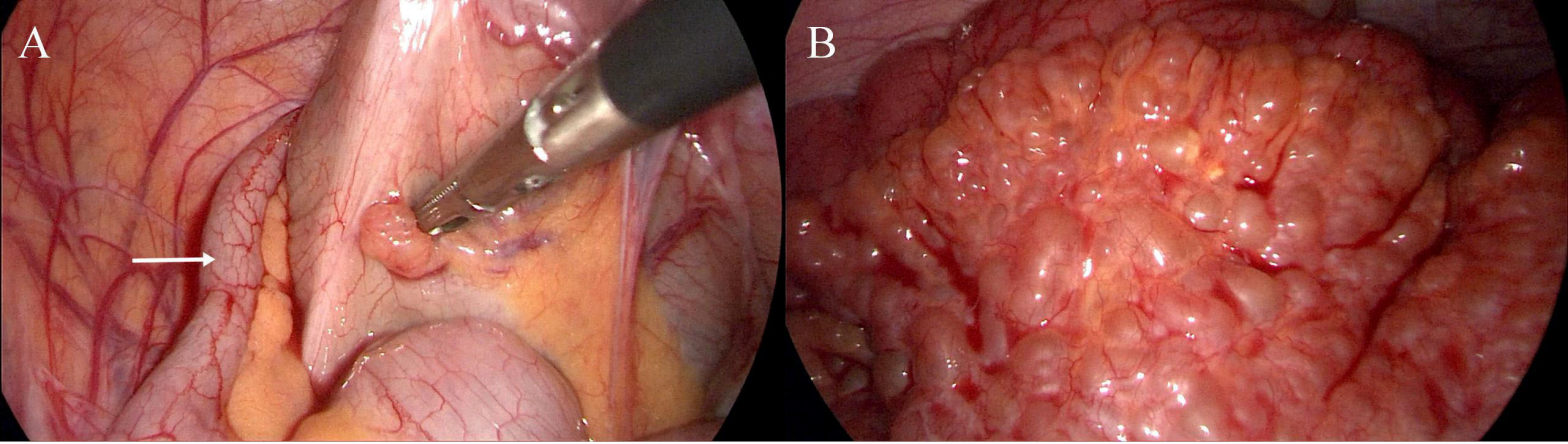
**(A)** The appendix was found to be approximately 10cm in length, 0.6cm in diameter, congested and edematous (white arrow); **(B)** Nodules of varying sizes are diffusely distributed throughout the mesentery of the small intestine.

**Figure 4:**
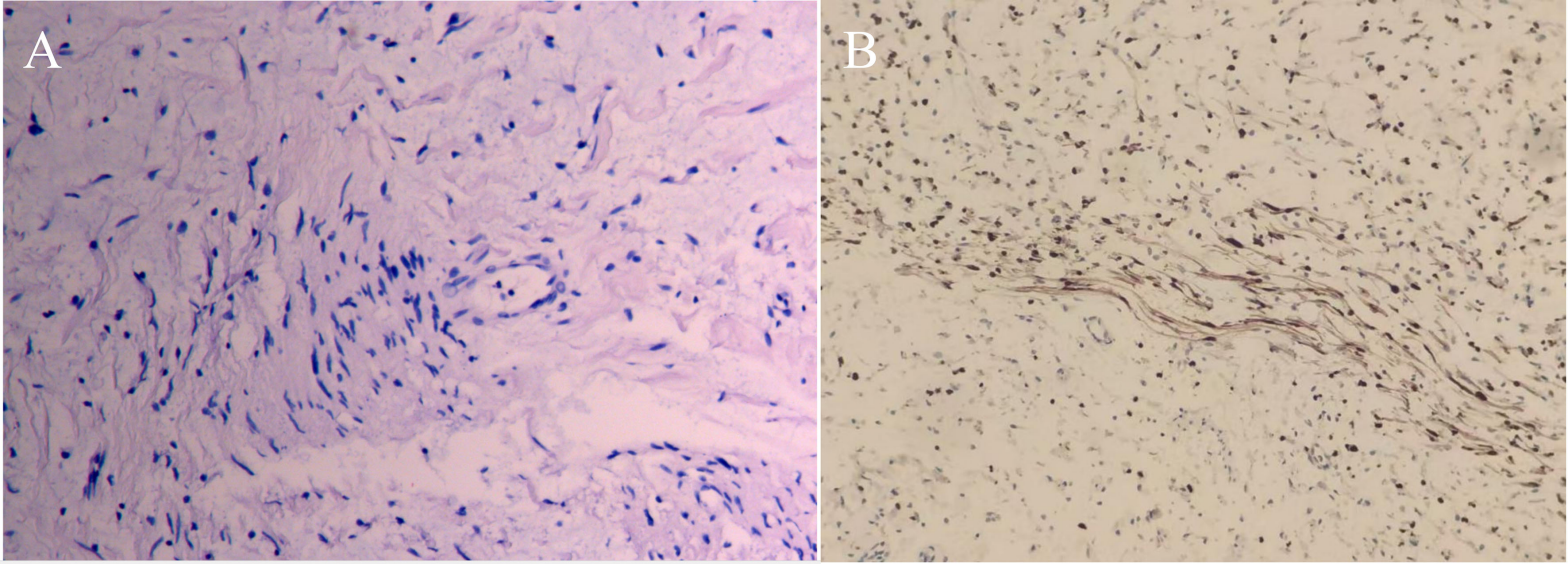
**(A)** Hematoxylin-eosin staining revealed a mesenteric neurofibromatosis (HE×40); **(B)** Immunohistochemistry suggests diffuse positivity of S-100 (×200).

The patient was initially diagnosed with acute appendicitis. In addition, considering the patient’s multiple café-au-lait spots and family history, neurofibromatosis was also considered. The patient underwent laparoscopic surgery, during which the appendix was found to be approximately 10cm in length, 0.6cm in diameter, congested and edematous, with mild adhesion to surrounding tissues (**Figure 3A**). The findings were consistent with acute appendicitis. More importantly, we observed diffuse nodules of varying sizes distributed throughout the small intestinal mesentery (**Figure 3B**), resulting in mesenteric stiffness, while the small intestinal surface remained unaffected. During surgery, we resected the mesenteric nodules and sent them for rapid pathological examination, which revealed interstitial fibrous tissue proliferation and edema, mucinous degeneration, and a tendency toward benign lesions. After communicating with the family, we performed appendectomy and mesenteric nodule biopsy. Based on our findings during surgery and the postoperative pathological diagnosis, the patient was ultimately diagnosed with acute appendicitis combined with mesenteric neurofibromatosis (**Figure 4**).

The patient was discharged on the fourth day without complications. One month after surgery, follow−up contrast−enhanced magnetic resonance imaging (MRI) of the brain and the entire spine was performed to evaluate for any central nervous system involvement. No abnormal intracranial or intraspinal lesions were detected. Currently, three months after surgery, telephone follow-up has revealed no abnormalities.

## 3 Discussion

Neurofibromatosis manifests in two primary types: Neurofibromatosis Type 1 (NF1), also known as peripheral neurofibromatosis, and Neurofibromatosis Type 2 (NF2), referred to as central neurofibromatosis. NF1 accounts for approximately 90% of all neurofibromatosis cases. This disorder predominantly affects the peripheral nervous system and skin. Its hallmark clinical features include café-au-lait spots, neurofibromas on peripheral nerves, Lisch nodules on the iris, and freckling in the axillary or inguinal regions. The presence of café-au-lait spots is one of the earliest indicators of NF1. These lesions appear as flat, hyperpigmented macules on the skin, typically oval-shaped. Furthermore, axillary or inguinal freckling-characterized by small freckles (<5mm) occurring in less common locations such as the axillae, groin, or neck-constitutes another significant diagnostic criterion for NF1 (1, 11). In the present case, physical examination revealed extensive café-au-lait spots covering the patient’s body. Combined with a significant family history, this finding strongly supported a diagnosis of neurofibromatosis.

Abdominal involvement in neurofibromatosis often presents with subtle and non-specific clinical manifestations. Research indicates that approximately 10-25% of Neurofibromatosis Type 1 (NF1) patients develop abdominal lesions (6, 9, 10). These lesions typically arise in the paravertebral region and retroperitoneal space (6). Mesenteric neurofibromatosis represents a rare manifestation of NF1. Clinical symptoms depend on the anatomical location of the lesions and the extent of mucosal involvement. Notably, about 65% of mesenteric neurofibromas remain asymptomatic. When symptomatic, clinical presentations may include a palpable abdominal mass, discomfort, or alterations within the affected nerve distribution territory (12). Histologically, these tumors consist of proliferating Schwann cells and fibroblasts embedded within a stroma rich in reticulin and collagen fibers, alongside a loose myxoid matrix (13). In the present case, the patient lacked overt clinical symptoms. The mesenteric involvement was incidentally identified during laparoscopic surgery performed for acute appendicitis, representing a clinically rare presentation.

Neurofibromatosis type 1 is also associated with a variety of central nervous system (CNS) abnormalities. Optic pathway gliomas (OPGs), typically low-grade pilocytic astrocytomas (WHO grade I), occur in approximately 15–20% of children with NF1 and may affect the optic nerves, chiasm, or tracts (14). Other low-grade gliomas can arise in the brainstem, cerebellum, or cerebral hemispheres (15). In addition, focal areas of signal intensity (FASIs)—previously termed unidentified bright objects (UBOs)—are frequently observed on T2-weighted and FLAIR sequences of unenhanced brain MRI in patients with NF1. These lesions are typically located in the basal ganglia, thalamus, cerebellum, and brainstem, and are generally considered benign and non-neoplastic (16, 17). Spinal cord involvement, including intramedullary low-grade gliomas, is less common (18). In the present case, postoperative contrast-enhanced MRI of the brain and the entire spine revealed no abnormal findings. No optic pathway glioma, other low-grade glioma, or FASI was detected. While our patient had no CNS involvement at the time of evaluation, these potential manifestations underscore the importance of routine neurological surveillance and baseline neuroimaging in patients with NF1.

A systematic literature review revealed no previously reported cases establishing a direct causal relationship between mesenteric neurofibromatosis and acute appendicitis. While appendiceal neurofibromas have been associated with diverticula formation and, theoretically, appendicitis through luminal obstruction or wall infiltration (19, 20), in our case the neurofibroma was confined to the mesentery without involvement of the appendiceal wall or lumen. Histopathological examination confirmed typical inflammatory changes of acute appendicitis in the appendix, distinct from the adjacent mesenteric neurofibroma. Therefore, we propose that the acute appendicitis was likely an incidental event in this patient with mesenteric neurofibromatosis, rather than a direct complication of the neurofibroma.

Diagnosis relies on a combination of genetic history, clinical presentation, and multimodal investigations including imaging, histopathology, immunohistochemistry, and chromosomal analysis. The clinical diagnosis of NF-1 is primarily based on the criteria established by the National Institutes of Health (NIH) Consensus Development Conference in 1987 (21). According to these criteria, an individual can be diagnosed with NF-1 if they meet two or more of the following features: (i) six or more café-au-lait macules; (ii) two or more neurofibromas or one plexiform neurofibroma; (iii) freckling in the axillary or inguinal region (Crowe’s sign); (iv) optic pathway glioma; (v) two or more Lisch nodules (iris hamartomas); and (vi) a characteristic osseous lesion. Moreover, if an individual has a first-degree relative with NF-1, only one of the above criteria is sufficient for diagnosis (22). More recent guidelines, such as those from the American Academy of Neurology, have reaffirmed these core criteria while incorporating updated genetic testing recommendations (23). In the present case, our patient exhibited multiple café-au-lait macules distributed across the trunk and extremities, diffuse mesenteric plexiform neurofibromas confirmed by histopathology, and a positive family history (her father also had characteristic cutaneous findings). Thus, the patient met the clinical diagnostic criteria for NF-1.

Imaging studies are also valuable for the diagnosis of NF1. On ultrasound, neurofibromas typically present as well-defined, homogeneous or heterogeneous hypoechoic masses (1, 24). Abdominal computed tomography (CT) offers particularly high diagnostic value for mesenteric neurofibromatosis. Most neurofibromas appear on non-contrast CT as smooth, rounded lesions with homogeneous low attenuation (approximately 20–30 Hounsfield units, HU) (12, 25). During the venous phase of contrast-enhanced CT, these lesions demonstrate moderate enhancement, ranging between 30–50 HU (1, 2, 6, 10). As proposed by Fukuya et al., these nodular densities represent cross-sectional images of enlarged peripheral nerves. Given their tendency to form interconnected networks, these nerves entrap normal peripheral adipose tissue within their plexiform architecture (26). The characteristic low attenuation observed radiologically correlates histologically with the tumors’ myxoid matrix (1, 3). Mesenteric neurofibromatosis invading the intestinal wall may manifest as submucosal or mucosal masses, or through extrinsic compression of the serosal surface by adjacent bowel involvement. Patchy or diffuse soft-tissue thickening represents a characteristic sign of neurofibromas penetrating the bowel wall (1). In this case, retrospective analysis of the patient’s preoperative lower abdominal CT revealed nodular mesenteric masses (**Figure 2B**). However, insufficient clinical recognition at the time precluded timely and accurate preoperative diagnosis. This underscores the imperative for heightened vigilance regarding potential mesenteric neurofibromatosis in NF1 patients undergoing abdominal surgery.

Histopathological examination remains the gold standard for diagnosing mesenteric neurofibromatosis. Both neurofibromas and schwannomas typically exhibit strong S-100 protein immunopositivity (13). In the present case, postoperative histopathology revealed strong S-100 positivity, confirming the diagnosis of mesenteric neurofibromatosis. PNs represent benign lesions in neurofibromatosis. However, their malignant transformation into malignant peripheral nerve sheath tumors (MPNSTs) constitutes a well-recognized complication associated with significant mortality in this patient population (27). Pathological examination is essential for distinguishing between these entities: Benign PNs demonstrate diffuse S-100 protein positivity, whereas MPNSTs typically exhibit negative or only focal S-100 immunoreactivity (28). Given the strong, diffuse S-100 positivity observed in this patient’s tumor, the findings are consistent with a benign plexiform neurofibroma. Consequently, conservative surveillance was deemed appropriate management at present.

The differential diagnosis of mesenteric spindle cell lesions in NF1 patients includes gastrointestinal stromal tumor (GIST), schwannoma, leiomyoma, and solitary fibrous tumor. GISTs, although rare in NF1, are KIT/CD117-positive and may arise in the small bowel mesentery (1). In contrast, neurofibromas are S-100-positive, CD34-negative, and lack KIT and DOG-1 expression. In this case, the tumor showed strong, diffuse S-100 positivity with negative staining for CD34, DOG-1, and SMA, effectively ruling out GIST, leiomyoma, and other mesenchymal neoplasms. The absence of SOX10 expression further supports the diagnosis of neurofibroma over schwannoma (29).

For mesenteric neurofibromatosis, complete surgical resection represents the primary therapeutic goal when feasible (6, 9, 30). In recent years, targeted therapy with mitogen-activated protein kinase kinase (MEK) inhibitors has revolutionized the treatment of inoperable or symptomatic plexiform neurofibromas (PNs) in NF−1 patients. The MAPK/ERK pathway is a key driver of NF1−associated tumorigenesis, and MEK inhibitors block downstream effectors of Ras, thereby inhibiting tumor growth (31). Selumetinib, a selective MEK1/2 inhibitor, was the first FDA−approved therapy for pediatric patients with symptomatic, inoperable PN (32). The pivotal phase 2 SPRINT trial demonstrated an objective response rate of 68% in children, with durable tumor volume reduction and significant pain improvement (32). Consequently, selumetinib has now been approved by the FDA for adults with NF1 and symptomatic, inoperable PN as well (32).

Mirdametinib, another MEK1/2 inhibitor, received FDA approval in 2025 for both adult and pediatric (aged ≥2 years) NF1 patients with inoperable PN, based on the phase 2b ReNeu trial, which reported objective response rates of 41% in adults and 52% in children (33). Given that complete surgical resection was not feasible in our patient due to diffuse mesenteric involvement, MEK inhibitor therapy could be considered as a potential treatment option in the event of future symptomatic progression or radiographic evidence of tumor growth. The availability of these targeted therapies offers a new paradigm for managing unresectable PN in both children and adults with NF−1, representing a major advance over previously available empiric agents.

## 4 Conclusion

Mesenteric neurofibromatosis is a rare, benign lesion with recognized malignant potential. Its frequent lack of specific clinical symptoms often leads to diagnostic confusion with other gastrointestinal disorders. Consequently, early screening for gastrointestinal involvement via imaging modalities is warranted in NF1 patients. Heightened clinical suspicion for mesenteric neurofibromatosis is particularly crucial for NF1 patients undergoing abdominal surgery. When identified early, complete surgical resection offers curative potential. In cases where complete excision is unattainable, protocol-based clinical and imaging surveillance is essential to monitor for early signs of malignant transformation.

## Data Availability

The original contributions presented in the study are included in the article/Supplementary Material. Further inquiries can be directed to the corresponding author.
